# Functional precision approach in patients with very high risk acute lymphoblastic leukaemia in India: a single-centre cohort study

**DOI:** 10.1016/j.lansea.2025.100710

**Published:** 2026-01-02

**Authors:** Jasmeet Sidhu, Arijit Chakraborty, Parag Das, Fabio D. Steffen, Subhajit Kundu, Sangramjit Basu, Bhaswati Tarafdar, Tanima Dey, Abhirupa Kar, Mousumi Biswas, Ankita Das, Naveen Sivadasan, Pritha Dasgupta, Niharendu Ghara, Beat Bornhauser, Jean-Pierre Bourquin, Shekhar Krishnan, Vaskar Saha

**Affiliations:** a Department of Paediatric Haematology and Oncology, Tata Medical Center, Kolkata, 700160, India; b Tata Translational Cancer Research Centre, Tata Medical Center, Kolkata, 700160, India; c Department of Oncology, University Children's Hospital Zurich, Zurich, 8008, Switzerland; d Division of Cancer Sciences, School of Medical Sciences, Faculty of Biology, Medicine and Health, University of Manchester, Manchester, M20 4BX, UK; e Tata Consultancy Services, Hyderabad, India

**Keywords:** Leukaemia, Childhood cancers, Very high risk, Precision oncology, Drug response profiling

## Abstract

**Background:**

Persistence of measurable residual disease (MRD) and high-risk cytogenetics are established predictors of relapse in childhood acute lymphoblastic leukaemia (ALL).

**Methods:**

Outcomes of children with ALL treated with the ICiCLe-ALL-2014 protocol at a single centre, between August 2013 and May 2023 were analysed. Co-culture *ex-vivo* drug response profiling (DRP) was performed on diagnostic or relapsed samples. Patients classified as very high risk (VHR) received DRP guided therapeutic modifications. Event free (EFS) and overall (OS) survival were compared across risk categories.

**Findings:**

Among 715 patients, at a median 55 (50–58) months, the 3-year EFS for standard-risk, intermediate-risk, high-risk B, T-ALL and VHR were 71% (64%–78%), 67% (58%–75%), 77% (70%–82%), 81% (71%–88%), and 38% (24%–52%) respectively (p < 0.0001). Persistent MRD at end of consolidation was associated with inferior EFS (40.3%, p ≤ 0.0001). Drug sensitivity scores from DRP performed on 112 samples identified panobinostat (median DSS 23.4), venetoclax (20.7), daunorubicin (17.9), selinexor (12.7) and bortezomib (12.1) as effective in VHR or relapsed ALL. From November 2020, 25 VHR patients received a modified treatment block incorporating venetoclax and bortezomib. At 1.5-year, landmark EFS was 81.8% (58%–93%) with the modified regimen vs 67.7% (49–81) with standard therapy (p = 0.0324). Venetoclax sensitivity correlated with MRD clearance (p = 0.0070).

**Interpretation:**

DRP enabled identification of effective agents for integration into therapy of VHR paediatric ALL. The addition of venetoclax and bortezomib was well tolerated and associated with improved early survival outcomes. These findings support prospective evaluation of DRP-guided treatment regimens in VHR ALL.

**Funding:**

DBT-Wellcome India Alliance, 10.13039/100012913Tata Consultancy Services.

**Research in context**

**Evidence before this study**

We searched PubMed (Jan 2010–May 2025) using “child”, “acute lymphoblastic leukaemia (ALL)”, “measurable residual disease (MRD)” and additional search terms of “relapsed”, “refractory”. Identified publications were further screened for “drug sensitivity testing” and “functional precision medicine”. In cooperative-group trials (COG, BFM, St. Jude Total, UKALL) persistence of MRD ≥0.01% after consolidation is predictive of relapse. Further intensification including allogeneic haematopoietic stem cell transplant does not significantly impact survival with 3-year event free survival (EFS) of <50%. In low-middle-income countries (LMICs), lack of immunotherapy and transplant access further worsens outcomes.

Image based *ex-vivo* drug screening was first introduced to individualise treatment for patients with acute myeloid leukaemia (AML). A functional precision medicine tumour board identified and successfully implemented individualised tailored therapies in relapsed/refractory AML patients. This approach is being investigated in a wider range of haematological malignancies in the EXALT 1 and 2 clinical trials. In paediatric ALL, DRP identified unique drug sensitivities across different genetic subtypes, offering genotypic individualisation of therapy. *KMT2A*-rearranged and early T-cell precursor ALL were BCL2-dependent and highly responsive to venetoclax. Subsequent clinical studies have shown a benefit of venetoclax-based regimens in patients with relapsed/refractory ALL.

**Added value of this study**

This study provides real-world evidence, from a LMIC, for functional precision medicine in treating children with ALL at a very high risk (VHR) of relapse. Analyses of outcomes of children with ALL treated uniformly showed a 3-year EFS of 38.1% for patients with persistent MRD or adverse cytogenetics. *Ex-vivo* drug response profiling (DRP) performed on bone marrow aspirates obtained prior to therapy, identified venetoclax, bortezomib, and panobinostat as the most active agents for VHR patients. DRP also revealed reproducible genetic subtype-specific vulnerabilities. Based on the findings from DRP, 25 VHR patients were treated with a modified delayed intensification (DI) block, substituting vincristine with bortezomib and adding venetoclax. This was well tolerated, with the 15 of 17 patients demonstrating a lowering of MRD. The landmark 1.5-year EFS of this group was 82% compared to 68% for VHR patients treated with the standard DI block (p = 0.0324).

**Implications of all the available evidence**

Taken together, emerging evidence from functional precision medicine studies demonstrates that *ex-vivo* DRP assays can be used to predict clinical sensitivity and integrated with therapeutic decision-making in childhood ALL. DRP-guided therapy modification is feasible within a LMIC network and can directly improve MRD clearance and short-term survival in children with VHR ALL. The identification of venetoclax and bortezomib as active agents against drug-tolerant blasts highlights the potential of integrating affordable, available and repurposed drugs into existing chemotherapy backbones. This approach provides a scalable framework for functional precision oncology in resource-limited settings, bridging discovery, implementation, and outcome measurement.

## Introduction

In acute lymphoblastic leukaemia (ALL) of childhood, risk stratified chemotherapy (based on genetic subtypes and measurable residual disease (MRD) and supportive care provides long-term survival rates of 90% in high-income countries (HICs). Persistence of MRD after 1–2 blocks of therapy and/or high-risk genetics (e.g. *KMT2A* rearrangements, hypodiploidy, *TCF3::HLF* fusion) identify patients with high probability of relapse despite intensifying treatment and allogeneic haematopoietic stem cell transplantation (allo-HSCT).^1^ The Children's Oncology Group reported a 5-year event free survival (EFS) of 43% (±7%) among children with detectable MRD at end of consolidation (EoC).^2^ St Jude's Total Therapy XV identified that patients with day 46 MRD ≥1% had 10-year EFS 56.4% despite intensive post-induction therapy.^3^ The use of allo-HSCT^4^ and/or targeted immuno/cellular therapies such as blinatumomab^5^ or CAR-T cells^6^ has shown promise in these patients. Their limited availability and high cost restrict widespread use in low- and middle-income countries (LMICs). In India, access to blinatumomab is currently limited to select centres under a humanitarian access program.^7^ Cellular and immunotherapies work best when MRD levels have been reduced using conventional cytotoxic therapy. We previously reported that *ex-vivo* drug response profiling (DRP) identified alternative active compounds, independent of somatic gene aberrations, in patients with suboptimal response to therapy.^8^ In this study, we describe a phenotypic precision approach to identify active therapeutic agents in a retrospective cohort of children with ALL who had poor outcomes with conventional risk-stratified therapy and were classified as very high risk (VHR). We further report on the application of this approach to treat a prospective cohort of VHR patients by incorporating agents identified through DRP in combination chemotherapy.

## Methods

### Study design and participants

Patients aged 1–18 years, newly diagnosed with ALL at our centre, treated with the ICiCLe-ALL-2014 (CTRI/2015/12/006434) protocol were eligible for the study.^9^ Sex was self-reported by participants or their guardians, with options limited to male and female. Patients with Ph-negative ALL (including ABL-class Ph-like ALL) and select adverse-risk genetics (*TCF3::HLF*,^10^ low hypodiploidy,^11^ infants with *KMT2A* rearrangements), or poor treatment response defined as >5% marrow blasts at end of induction (EoI), MRD ≥1% at EoI or detectable MRD at end of consolidation (EoC) or a combination of the two (*IKZF*^plus^^12^ with EoI MRD ≥0.01%) were eligible for alternative therapy and/or allo-HSCT and categorised as VHR for the purposes of this paper. From November 2020, blinatumomab became available for select B-cell precursor (BCP) ALL patients under a humanitarian access program.

### Procedures

Serial bone marrow aspirates and/or peripheral blood samples were obtained from patients with initial or relapsed ALL after obtaining consent. For image-based DRP, 2.5 × 10^3^ h-TERT immortalised mesenchymal stromal cells (MSCs) were seeded per well in 384 well plates, 24 h before adding 1–2.5 × 10^4^ primary ALL cells/well. Stock solutions for all drugs used in DRP were prepared at 5 mM concentration in DMSO, except for asparaginase, which was reconstituted in fresh frozen plasma (FFP) to a concentration of 500 IU/mL, and were stored at −80 °C. On Day 3 of assay (after 24 h of co-culture incubation at 37 °C, 5% CO_2_), serial dilutions of compounds in the drug panel (Supplemental Table S1) were prepared in AIM-V media and added in triplicates (10 μL per well). Automated imaging was performed 72 h after drug addition using high-throughput microscope (Molecular Devices) with 10× Plan Fluor. Drug response curves were generated for live cell count using 4-parameter log-logistic function and normalised against untreated control wells. Drug response was evaluated as drug sensitivity scores (DSS) based on area–under curve.^13^ Details are provided in the Supplemental Methods.

### Statistical analyses

Patient characteristics and treatment responses were compared using two-tailed Fisher's exact test. Survival analyses were performed using Kaplan–Meier method, and differences in EFS and OS assessed using two-tailed log-rank test. EFS was defined from the start of final risk stratification (day 35) until treatment-related death, non-remission/disease progression, or relapse. OS was defined from the start of final risk stratification until death from any cause. For landmark survival analysis, EFS was defined from the start of the delayed intensification (DI) phase until disease progression, relapse, or treatment-related death. Treatment-related toxicities were captured using the Common Terminology Criteria for Adverse Events (CTCAE) version 5 guidelines. Proportions of CTCAE Grade 3–5 toxicity incidences were compared using a two-tailed Fisher's exact test. All analyses were conducted using R version 4.4.1, SPSS 25, Stata BE18, and GraphPad Prism version 10. This report follows the STROBE reporting guidelines^14^ for cohort studies and the checklist is available in the supplementary data file.

### Ethics statement

This study received the following ethical approvals from the Institutional Review Board of the Tata Medical Centre. EC/TMC/12/13 October 28th 2013; EC/TMC/12/13, revised January 31, 2019; EC/TMC/65/16, February 19, 2019 and 2019/Govt/28/IRB51, September 27, 2019 for treatment, biobanking, biomarker analyses and DRP respectively. Written consent was obtained from parents or legally authorised surrogates, and assent was sought for participants aged ≥8 years, for treatment, biobanking and laboratory analyses.

### Role of the funding sources

The study's funding sources were not involved in the research design, data collection, analysis, interpretation or writing of the report.

## Results

**Fig. 1:**
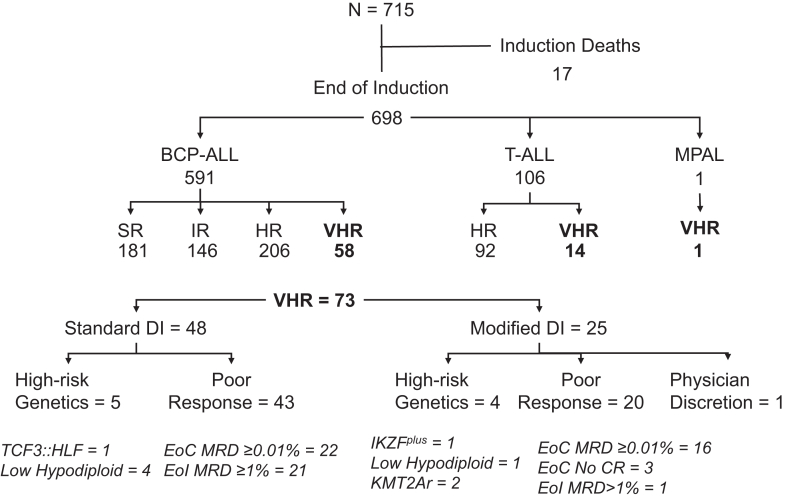
**Patient risk stratification and treatment assignment for children with acute lymphoblastic leukaemia (ALL)**. Flow diagram illustrating risk stratification for 715 children diagnosed with ALL.

Between August 2013 and May 2023, 715 children with ALL were sequentially treated at our centre using the risk-stratified ICiCLe-ALL-2014 protocol, including 106 (15%) with T-ALL (Fig. 1). Induction mortality was 2.4% (17/715). Post-induction, 181 (25%), 146 (21%), and 206 (30%) BCP-ALL patients were classified as standard-risk (SR), intermediate-risk (IR) and high-risk (HR) respectively. Additionally, 73 patients (10%) were categorised as VHR, including 48 treated with the standard ICiCLe-ALL-14 protocol (until October 2020), and 25 whose post-induction treatment was subsequently modified. Patient characteristics are summarised in Table 1.

**Table 1:** Clinical characteristics of patient cohort.

N	715	%
**Sex**		
Male	450	63
Female	265	37
**Age at diagnosis (years)**		
<10	561	78
≥10	154	22
Median	5	
Interquartile range	3.2–9.2	
**hWCC (×10^9^/L)**		
<50	519	73
≥50	188	26
Not available	8	1
**Lineage**		
B	605	85
T	109	15
Mixed	1	
**Cytogenetic group**		
**Good risk**		
*ETV6::RUNX1*	115	16
High hyperdiploidy	226	32
**Intermediate risk**		
B-others	175	24
*TCF3::PBX1*	31	4
**High risk**		
Hypodiploidy	5	1
*TCF3::HLF1*	1	0
*KMT2A* rearranged	17	2
iAMP21	4	1
*BCR::ABL1*	15	2
T-ALL	106	15
Unknown	20	2
**CNS status**		
CNS1	677	95
CNS2	11	2
CNS3	16	2
Not available	11	2
**Prednisolone Response**		
Good	515	72
Poor	72	10
Not evaluable	73	10
Abrogated	55	8
**MRD at EoI (%)**		
<0.01	475	66
≥0.01	165	23
Unknown^a^	75	10
**MRD at EoC (%)**		
<0.01	519	73
≥0.01	47	7
Unknown^b^	149	21
**Final risk**		
Standard	181	25
Intermediate	146	20
High (includes T)	298	42
Very high	73	10
Not evaluable	17	2

VHR, very high risk; hWCC, highest presenting white cell count; IQR, inter-quartile range; MRD, minimal residual disease; EoI, end of induction; EoC, end of consolidation.

a Includes 17 induction deaths.

b Includes 17 induction and 4 consolidation deaths.

At a median of 54.5 months (95% CI: 50–58), the 3-year EFS and OS for SR, IR, HR, T-ALL and VHR were 71.3% (95% CI: 64–78), 67.1% (58–75), 76.7% (70–82), 80.9% (71–88) and 38.1% (24–52) (logrank, p < 0.0001); and 94.0% (89–97), 78.1% (70–84), 85.7% (80–90), 85.0% (76–91), and 57.4% (41–71) (log-rank p < 0.0001, respectively (Supplemental Figure S1, Supplemental Table S2). Children diagnosed with VHR ALL showed markedly poorer outcomes than those with non-VHR ALL, with EFS of 38.1% (24–52) vs 73.3% (69–77; p < 0.0001) and OS of 57.4% (41.1–70.8) compared to 86.3% (83.2–88.9; p < 0.0001) (Supplemental Figure S2).

Among the 48 VHR patients, there were 2 treatment related deaths (TRD), 7 experienced progressive disease and 25 relapsed. Seventeen (50%) of these 34 events occurred within the first 12 months, and 21 (62%) within 18 months of treatment initiation. All patients in the VHR group were recommended allo-HSCT, but none were transplanted, mostly due to financial constraints. At EoC, 33 (69%)/48 patients had evidence of residual disease. These included 22 patients with MRD ≥0.01% both at EoI and EoC, and 11 who were non-CR or MRD ≥1% at EoI as well as non-CR or MRD ≥0.01% at EoC. At EoC, 6 had marrow blasts ≥5%, 25 had MRD ≥0.01%, and 2 had persistent extramedullary disease. Of the 33 patients with disease at EoC, 17 relapsed, 5 had progressive disease, and 1 had TRD during the maintenance phase.

As majority of VHR patients had persistent disease at EoC, we analysed the impact of MRD clearance rates on survival in 537 patients with available MRD values at both EoI and EoC. Patients were stratified into three MRD groups: Group 1 (MRD <0.01% at both EoI and EoC), Group 2 (MRD ≥0.01% at EoI and <0.01% at EoC) and Group 3 (MRD ≥0.01% at both time points). Compared to the Group 1, patients with Group 3 had a significantly higher incidence of hyperleukocytosis at diagnosis (white cell count ≥50,000/μL: 43.3% vs 23.5%, p = 0.0243), and greater prevalence of high-risk cytogenetics (16.7% vs 4.8%, p = 0.0203) (Supplemental Table S3). No significant differences were observed in the proportion of older patients (age ≥10 years) or those with poor prednisolone response. The 3-year EFS was 71.6% (66.9–76.7) for Group 1, 75.0% (66.1–85.2) for Group 2 and 40.3% (25.6–63.5) for Group 3 (log-rank p < 0.0001, Supplemental Figure S3). On multivariable cox proportional hazards analysis, using Group 1 as the reference, patients in Group 3 had a significantly increased risk of adverse events (relapse or death) (hazard ratio 3.65; 95% CI: 2.29–5.82; p < 0.001). In contrast, Group 2 did not show a statistically significant difference in risk compared to the Group 1 (hazard ratio 1.20; 95% CI: 0.81–1.78; p = 0.364) (Supplemental Table S4).

The persistence of MRD and high rate of recurrence of disease while still on therapy suggests that VHR patients have a population of cells tolerant to the drugs used in the first few weeks of therapy.^15^ To investigate this DRP^8^ was performed on cryopreserved or fresh MNCs from 112 blast-enriched bone marrow aspirate samples obtained prior to start of therapy in 72 (64.3%) newly diagnosed and 40 (35.7%) relapsed ALL patients (Supplemental Table S5). Ninety-five samples were from BCP-ALL, 15 T-ALL and 2 mixed phenotype acute leukaemia (MPAL). Drugs analysed were prednisolone, vincristine, daunorubicin, asparaginase, cytarabine, cyclophosphamide, 6-thioguanine (the active metabolite of 6-mercaptopurine) and mitoxantrone, used in newly diagnosed ALL^9^; bortezomib, used in relapsed ALL^16^; and panobinostat, venetoclax, selinexor, three novel agents with reported activity in ALL, available to us at that time.

**Fig. 2:**
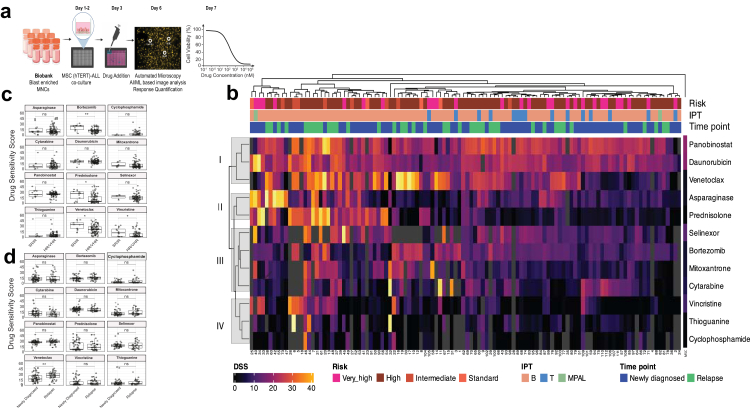
***Ex-vivo* drug response profiling reveals heterogeneous sensitivity patterns across clinical subgroups in ALL**. (a) Schematic representation of the drug response profiling (DRP) workflow. (b) Hierarchical clustering heatmap of DSS values across 112 patient samples and 12 drugs. (c) Comparison of DSS between group 1 (SR/IR) and group 2–4 (HR/VHR/relapse). Boxplots display DSS distribution for each drug. (d) Comparison of DSS between newly diagnosed and relapsed ALL patients. Boxplots show drug-wise DSS variation across the two time-points. p < 0.05 considered significant. ^∗^indicates p < 0.05; ^∗∗^indicates p < 0.01, ns indicates not significant differences.

Sample processing and analyses are summarised in Fig. 2a. Primary samples had a mean (±SD) blast percentage of 80% (±18%) with mean (±SD) pre-seeding viability of 85% (±14%). hTERT-immortalised MSCs were tested separately to investigate the effect of dose ranges used and remained viable at all concentrations. Unsupervised clustering of DSS identified four major clusters (Fig. 2b). Cluster I included panobinostat (pan-histone deacetylase inhibitor, median DSS 23.5), venetoclax (BCL-2 inhibitor, median DSS 20.2), and daunorubicin (topoisomerase II inhibitor, median DSS 19.8), with a cluster median DSS of 21.9 (Table 2). Cluster III showed moderate sensitivity with wider variability and included selinexor (selective exportin-1 inhibitor, median DSS 13.2), bortezomib (proteasome inhibitor, median DSS 12.6), mitoxantrone (anthracycline, median DSS 7.4), and cytarabine (nucleoside analogue, median DSS 7.6), with a cluster median DSS of 11.1. Cluster II included asparaginase (median DSS 9.7) and prednisolone (median DSS 6.9) and demonstrated selective sensitivity in a subset of patients. Cluster IV (median DSS 1.05) included cyclophosphamide, 6-thioguanine, and vincristine which demonstrated minimal activity across most samples.

**Table 2:** Median drug sensitivity scores for select agents in each drug response cluster.

	Cluster DSS			Per drug DSS	
	Median	IQR		Median	IQR
Cluster I	21.9	16.1–25.8	Panobinostat	23.5	21.6–26.5
			Daunorubicin	19.8	16.2–23.5
			Venetoclax	20.2	11–27.4
Cluster II	8.6	3.6–17.4	Asparaginase	9.7	5.8–16
			Prednisolone	6.9	2.6–20.9
Cluster III	11.1	5.7–15.2	Selinexor	13.2	8.9–17.6
			Bortezomib	12.6	9.3–14.4
			Mitoxantrone	7.4	5–13.1
			Cytarabine	7.6	4–14.2
Cluster IV	1.05	0–7.3	Vincristine	1	0–8
			Thioguanine	2.1	0–6.1
			Cyclophosphamide	0	0–3.5

**Fig. 3:**
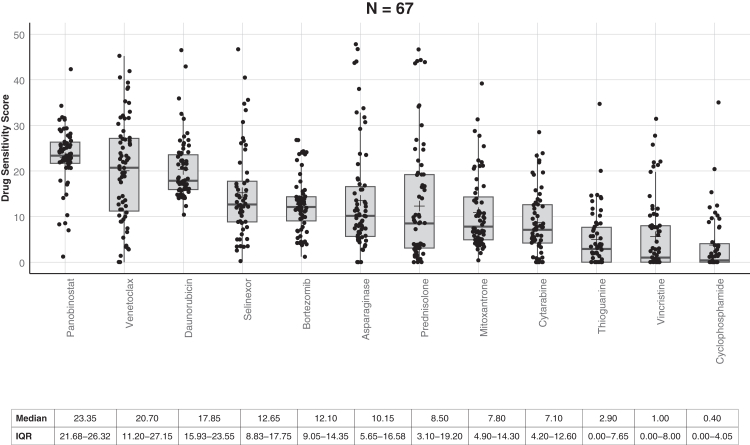
***Ex-vivo* drug sensitivity in samples from patients with Relapse/VHR ALL.** Boxplots showing Drug Sensitivity Scores (DSS) across 67 samples from patients with relapsed/VHR ALL. Each dot represents an individual sample.

To correlate this with the observed clinical response, results were grouped as follows: (1) newly diagnosed, treated as SR (n = 4) and IR (n = 8), prednisolone good responders and MRD <0.01% at EoI, i. e the most chemosensitive; (2) newly diagnosed, treated as HR and/or prednisolone poor responders and/or MRD ≥0.01% at EoI (n = 27) or T-ALL (n = 5) i.e. moderately drug tolerant; (3) VHR as previously defined (n = 27), with a high probability of recurrence and (4) patients with documented relapse (B-ALL 38, T-ALL 2), 34 in first relapse and 6 in second relapse. Group 1 patients showed significantly higher *ex-vivo* sensitivity for prednisolone (p = 0.0164) and vincristine (p = 0.0490) compared to those in groups 2–4 as expected and as previously reported^17^ (Fig. 2c, Supplemental Table S6). The median DSS for prednisolone and vincristine were 26.1 and 10.4 in Group 1 vs 4.1 and 0.7 in groups 2–4, respectively. Group 1 also demonstrated significantly greater sensitivity to bortezomib (median DSS: 24.3 vs 12.2; p = 0.0051) and venetoclax (median DSS: 33 vs 19.5; p = 0.0106). No significant differences in DSS were observed for daunorubicin and asparaginase across risk groups. Venetoclax was the only drug which demonstrated significantly higher sensitivity in Group 4 (relapse ALL) as compared to Group 1–3 (newly diagnosed) (median DSS 25.9, IQR 19.3–31.6 vs 15.3, IQR 9–24.6; p value 0.0027) (Fig. 2d). Samples with T-ALL exhibited similar sensitivity profiles to those with B-ALL, except for prednisolone and venetoclax, both of which were significantly more active in B-lineage disease, with p-values of 0.0311 and 0.0144, respectively (Supplemental Figure S4). The median DSS for prednisolone and venetoclax were 3.3 and 12.1 in T-ALL and 8.7 and 21.9 in B-ALL respectively. Data was combined for VHR and relapsed ALL which represent the more drug tolerant groups. These samples were most sensitive *ex-vivo* to panobinostat (median DSS 23.35), venetoclax (DSS 20.7) followed by daunorubicin (DSS 17.85), selinexor (DSS 12.65), and bortezomib (DSS 12.1) (Fig. 3). In contrast, these samples were tolerant to drugs forming the backbone of current chemotherapy i.e. prednisolone, cytarabine, 6-thioguanine, vincristine, and cyclophosphamide, with DSS of 8.5, 7.1, 2.9, 1 and 0.4 respectively.

The distribution of DSS across cytogenetic subtypes of ALL revealed distinct subtype-specific patterns (Supplemental Figure S5A). Most subtypes were tolerant to vincristine, cyclophosphamide and thioguanine. All cytogenetic groups analysed were consistently sensitive to panobinostat. As reported earlier,^17^ asparaginase demonstrated significantly greater sensitivity in *ETV6::RUNX1* samples with a median DSS of 16.9 as compared to 8.7 in other subtypes combined (p = 0.0043) (Supplemental Figure S5B). *TCF3::PBX1* samples (n = 5, 4 relapsed, 1 newly diagnosed) were sensitive to bortezomib with a median DSS of 24.3 as compared to 12.5 in other subtypes (p = 0.0450) (Supplemental Figure S5C). The sole low hypodiploid sample was sensitive to venetoclax (DSS 31.5)^18^ and selinexor (DSS 33.3). The DSS patterns of 12 samples with VHR cytogenetics (2 with *IKZF*^plus^, 9 with *KMT2A* rearrangement, 1 with low hypodiploidy) were most sensitive to daunorubicin (median DSS 22.8, IQR 17.9–26.3) and panobinostat (22.5, 22.1–24) followed by venetoclax (13.2, 8.5–27.1) and selinexor (13.1, 11.2–23.6), though with higher inter-sample variability (Supplemental Figure S5D). Therefore, panobinostat, venetoclax, bortezomib and selinexor were identified as potential novel agents for treatment of high-risk genetic subtypes, including relapsed *TCF3::PBX1* as well as for patients with poor response to therapy.

**Fig. 4:**
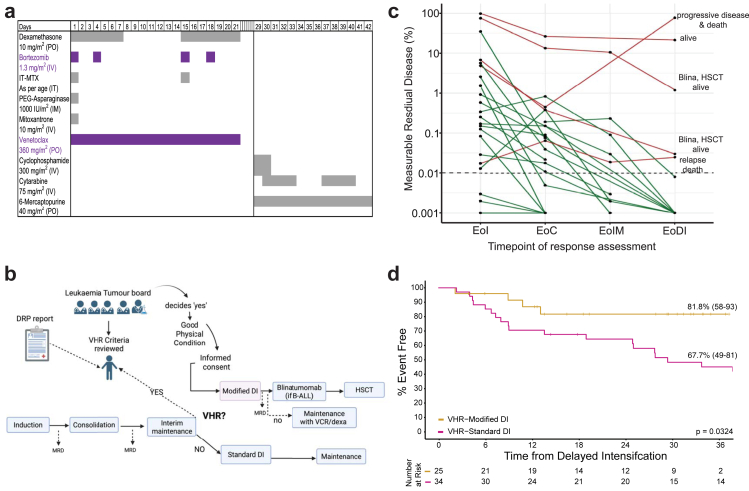
**Modification of delayed intensification (DI) block based on findings of drug response profiling improves response and survival rates in VHR ALL**. (a) Schematic of modified DI block. Agents highlighted in purple are the new drugs. (b) Decision-making algorithm for eligibility for modified DI. VHR criteria, general condition, and DRP results were reviewed by a leukaemia tumour board. (c) Longitudinal MRD response in VHR patients treated with modified DI. (d) Landmark analysis comparing event free survival (EFS) between modified DI and standard DI in VHR ALL.

In the ICiCLe protocol, post EoC, HR patients receive high-dose methotrexate based interim maintenance followed by a DI block comprising vincristine, asparaginase, anthracycline (mitoxantrone or doxorubicin), dexamethasone, cyclophosphamide, cytarabine and 6-mercaptopurine. DRP suggested that venetoclax, bortezomib, panobinostat, and selinexor are active in samples of VHR ALL. We have previously reported the successful substitution of vincristine with bortezomib, and its use in combination with asparaginase, mitoxantrone, and dexamethasone to induce second remission in patients with relapsed ALL.^16^ Venetoclax has shown promising efficacy and tolerability in adult ALL,^19^^,^^20^ and its use in children has been reported, including dose and schedule guidance.^21^^,^^22^ A recent report further suggests that venetoclax can potentiate the activity of asparaginase in drug tolerant cells.^23^ Panobinostat is no longer accessible for our patients. Based on this, the DI of ICiCLe was modified by replacing 4 doses of vincristine with 4 doses of bortezomib (1.3 mg/m^2^ each), increasing the dexamethasone dose from 6 mg/m^2^/day to 10 mg/m^2^/day (2 pulses, 7 days each) and adding venetoclax (360 mg/m^2^/day, capped at 400 mg/day) for 21 days. Antifungal and antibacterial prophylaxis with liposomal amphotericin B (2.5 mg/kg/dose, twice weekly, capped at 100 mg/dose) and trimethoprim-sulfamethoxazole was administered throughout the block (Fig. 4a). Cyclophosphamide was given in fractionated doses, as reported previously.^24^ Eligibility for modified DI block was determined by the leukaemia tumour board, based on VHR criteria, DRP data (where available) and clinical condition. Patients were counselled and offered modified or standard DI after written informed consent (Fig. 4b). All 25 patients classified as VHR (4 VHR genetics, 20 poor responses to therapy, 1 physician discretion) by the tumour board (Fig. 1) between November 2020 and May 2023 opted for modified DI. BCP-ALL patients were offered blinatumomab and all patients offered allo-SCT. Patients who received modified DI were comparable in key prognostic characteristics, including age, sex, immunophenotype, presenting white-blood-cell count, cytogenetics, and MRD response with the retrospective VHR cohort (Supplemental Table S7).

Of the 25 patients, 20 were BCP-ALL and 5 T-ALL. Five BCP-ALL patients received two cycles of blinatumomab after modified DI, of whom three proceeded to allo-HSCT (Supplemental Figure S6A). Patients who did not receive allo-HSCT received maintenance therapy with 6-mercaptopurine and methotrexate with addition of monthly vincristine-dexamethasone pulses, and an additional year of maintenance in *IKZF1*-deleted ALL.^25^ Of 20 patients with poor response to therapy, 17 had detectable MRD at EoC, 2 had isolated persistent extramedullary disease at EoC and 1 had induction failure with MRD <0.01% at EoC. At end of modified DI, two patients with persistent extramedullary disease remain in remission. Of the 17 with detectable MRD at EoC, 15 demonstrated a declining MRD trend with modified DI (Fig. 4c). One patient with BCP-ALL, characterised by CNS2 status, *TCF3::PBX1*, poor prednisolone response on day 8, and M3 marrow at EoI, exhibited progressive disease during therapy and subsequently died. Among the four patients who had MRD ≥0.01% following modified DI, two proceeded to receive blinatumomab followed by allo-HSCT, while the remaining two were treated with maintenance chemotherapy; of these, one relapsed and died. Overall, of 25 patients treated on modified DI, 21 remain in CR1, 3 relapsed and 1 had progressive disease (Supplemental Figure S6A).

The modified DI block was well tolerated, with no statistically significant increase in CTCAE grade ≥3 toxicities compared to retrospective VHR cohort treated with the standard DI block (Table 3). While a higher proportion of modified DI recipients experienced grade 3 events, primarily febrile neutropenia, these did not progress to life-threatening illness or result in mortality.

**Table 3:** Toxicity profile in VHR patients treated with standard vs modified delayed intensification.

Toxicity grade	Standard DI		Modified DI		p
N	34		25		
NCI-CTCAE	n	%	n	%	
**Grade 3**	17	50	18	72	0.112
Fever neutropenia	12		13		
Vomiting	2				
Chemical meningitis	2				
Asparaginase hypersensitivity	1		1		
Pancreatitis			1		
Hyperglycaemia			2		
Peripheral neuropathy			1		
**Grade 4**	3	9	4	16	0.443
Fever neutropenia with sepsis	3		1		
Neutropenic enterocolitis			2		
**Grade 5**	0	0	0	0	1

Landmark survival analysis from the start of DI demonstrated 1.5-year EFS of 81.8% (95% CI 58.2–92.8) in modified DI cohort, compared to 67.7% (95% CI 49.2–80.6) in those who received standard DI block (p = 0.0324; Fig. 4d). Five patients who received two cycles of blinatumomab, of whom three proceeded to allo-HSCT, remain disease-free at a median follow-up of two years (Supplemental Figure S6B).

**Fig. 5:**
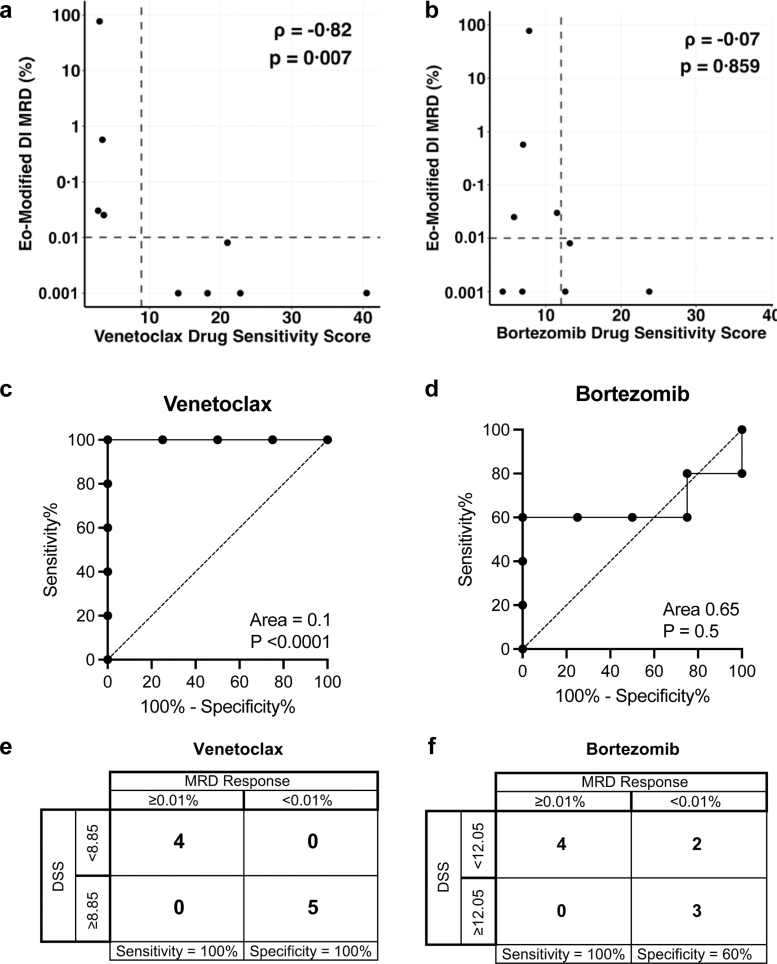
**Correlating *ex-vivo* drug sensitivity (venetoclax, bortezomib) with clinical response to modified delayed intensification**. (a, b) Correlation between Drug Sensitivity Scores (DSS) and end-of-modified DI (Eo-Modified DI) MRD levels. (c, d) Receiver operating characteristic (ROC) curves evaluating DSS as a predictor of MRD response. (e, f) Contingency matrices comparing DSS-based classification with clinical MRD response.

DRP was done at diagnosis for 9 of 25 patients who received modified DI block. All 9 patients had detectable MRD at EoC. Five had MRD <0.01% at the end of the modified DI and 4 had MRD ≥0.01% including one who failed to achieve CR. The DSS of venetoclax and bortezomib, the two novel agents in modified DI, were correlated with the MRD response. A strong inverse correlation was observed between venetoclax DSS and MRD levels (Spearman's ρ = −0.82, p = 0.0070), with higher DSS associated with lower MRD (Fig. 5a). In contrast, the correlation between bortezomib DSS and MRD was not statistically significant (ρ = −0.07, p = 0.859) (Fig. 5b).

ROC analysis identified DSS thresholds to discriminate MRD-negative (<0.01%) and MRD-positive (≥0.01%) patients. For venetoclax, DSS cut-off of <8.85 perfectly discriminated MRD-positivity from MRD negativity with 100% sensitivity (95% CI 51.0–100) and 100% specificity (95% CI 56.6–100), AUC of 1.0 (95% CI 1.00–1.00, p < 0.0001, DeLong) (Fig. 5c). In comparison, bortezomib demonstrated DSS threshold of <12.05, which maintained 100% sensitivity (95% CI 51.0–100%) but 60% specificity (95% CI 23.1–88.2%), and an AUC of 0.65 (p = 0.5, DeLong) (Fig. 5d). Contingency analysis showed that all MRD-negative patients were classified as sensitive and all MRD-positive patients as resistant to venetoclax (Fig. 5e). For bortezomib, the DSS threshold correctly identified MRD positivity, but misclassified two MRD-negative patients as resistant (Fig. 5f).

## Discussion

In this study we report outcomes of 715 children with ALL treated on the ICiCLe-ALL-2014 protocol at a single centre, focussing on the VHR subgroup. Real-world data confirmed the poor outcomes of this subgroup. Nearly 70% of VHR patients had persistent MRD at EoC, with most relapsing while still on treatment, leading to a dismal 3-year EFS (38.1%) and OS (57.4%). Due to resource constraints, few underwent allo-HSCT. Access to blinatumomab and CAR-T remains limited and expensive. Early phase investigational drugs, de rigueur in HICs, are largely unavailable. Presence of MRD at EoC reflects persistent drug tolerant cells,^15^ independent of genotype^26^ underscoring the limitation of conventional chemotherapy to achieve durable remission. DRP provided critical insights into *ex-vivo* chemosensitivity patterns across risk groups and cytogenetic subtypes. Panobinostat, venetoclax and bortezomib retained high DSS in VHR and relapsed samples, while standard chemotherapeutics like vincristine and cyclophosphamide showed limited activity. Venetoclax showed greater sensitivity in relapsed ALL samples and in high-risk genetics, aligning with emerging data supporting its incorporation into refractory paediatric leukaemia protocols.^19^^,^^27^ Asparaginase sensitivity varied and resistance can be modulated by co-targeting BCL2^23^ or the mitochondrial redox system.^28^ Similarly bortezomib potentiates the effect of dexamethasone, particularly in T-ALL.^29^ Overall combining BCL2, proteasome and histone deacetylase inhibition with asparaginase could overcome drug tolerance in VHR patients.

VHR patients are identified after first two therapy blocks, when they are eligible for alternative therapy and allo-HSCT. The design of DI block allowed integration of venetoclax and bortezomib for VHR patients, yielding encouraging outcomes. Compared to retrospective cohort treated with standard DI, patients receiving modified DI had superior 1.5-year EFS (81.8% vs 67.7%, p = 0.032) without excess grade ≥3 toxicities. The two groups are not directly comparable as over time patients benefitted from better quality asparaginase^30^ and more sensitive MRD assays.^31^ Nevertheless our observations identify a potential strategy for VHR patients in LMICs where therapeutic options are limited. A subset of patients bridged to blinatumomab and allo-HSCT achieved durable remissions, supporting its role both as stand-alone intensification and as a bridge to definitive therapy (Supplemental Figure S6B).

These findings suggest *ex-vivo* sensitivity testing as a valuable tool to stratify patients likely to benefit from targeted chemotherapy modifications. The new drug combination reduced MRD and improved early survival, with manageable safety. The strong inverse correlation between venetoclax DSS and post-DI MRD (ρ = −0.82, p = 0.0070) supports the clinical relevance of DRP in predicting treatment response, potentially sparing toxicity in unlikely responders. Distinct cytogenetic drug sensitivity patterns were evident: *ETV6::RUNX1* showed asparaginase sensitivity, *TCF3::PBX1* had bortezomib sensitivity, and *KMT2A* rearranged samples were consistently sensitive to daunorubicin, panobinostat, and venetoclax. These subtype-specific vulnerabilities could inform future risk-adapted regimens.

While findings are promising, limitations remain. Few patients received modified DI and had DRP analysed prior to treatment, limiting generalisability. The numbers studied are small, MRD response was not available for all patients. The correlation of venetoclax DSS as a predictor of MRD response requires further validation as a tool for individualising therapy. Though early MRD responses and 1.5-year EFS are encouraging, whether these responses translate into durability of remissions, particularly in patients who did not receive immunotherapy or allo-HSCT, remains to be seen. DRP evaluated 12 agents, unlike other studies that assessed larger drug panels specific for T and BCP-ALL. This focused approach was intended to prioritise agents with repurposing potential in our setting. We plan to scale up the number of chemotherapeutic agents in DRP to identify more drugs that can be repurposed. Additionally, DRP analyses were limited to a subset of patients. Prospective validation in larger, multi-centre cohorts is essential to establish its predictive value for real-world outcomes.

Recent single-cell studies show that leukaemia cells with haematopoietic stem and progenitor cell (HSPC)-like transcriptional states are associated with therapy resistance and persist after induction.^26^ These HSPC-like blasts are resistant to conventional cytotoxics, exhibit elevated BCL2 expression, and are enriched in patients with higher MRD. These cells are sensitive to venetoclax, aligning with our findings. In this context, BH3 profiling could further refine patient stratification by directly measuring BCL2 dependence at baseline, identifying those harbouring chemo-tolerant HSPC-like blasts likely to benefit from venetoclax.^32^ Analysing BH3 mimetics with DRP may inform the rational design of next-generation therapeutics, including PROTACs (Proteolysis Targeting Chimeras), targeting specific anti-apoptotic proteins in future precision medicine strategies.

In conclusion, our findings demonstrate that *ex-vivo* phenotypic drug profiling can identify vulnerabilities in VHR ALL. Incorporating novel agents such as venetoclax and bortezomib during DI improves MRD clearance and short-term survival. These results support the integration of DRP-guided, risk-adapted therapies in resource-limited settings to optimise outcomes for children with high-risk ALL, now possible with the ICiCle network. For this it will be important to develop a standardised and reproducible DRP assay. Future studies could involve the prospective banking of diagnostic bone marrow aspirates and performing DRP for patients with suboptimal treatment response. Further studies combining these approaches with immunotherapies and strategies to improve transplant access are warranted to achieve durable remissions in this challenging population.

## Contributors

Conceptualization: JS, SK, VS; DRP experiments: AC, AD, TD; Biobanking of samples: SK, AK; Data Curation: MB, PD, AC; Formal Analysis: JS, PD; Visualization: SB, NS, FS; Writing—Original Draft: JS; Writing—Review & Editing: JS, VS, JP, BB; Supervision: VS; Clinical Management: NG, VS, JS; Funding Acquisition: JS, VS. JS and VS have directly accessed and verified the underlying data reported in the manuscript. All authors reviewed and approved the final version of the manuscript.

## Data sharing statement

The data underlying the results reported in this paper will be available, after deidentification, from the corresponding authors on reasonable request. Data will be provided to researchers who provide a methodologically sound proposal to enhance the aims of the study, approved by an independent review board.

## Declaration of interests

Authors do not have any conflicts of interest to declare.

## Appendix A Supplementary data

Supplemental Data

Treatment Protocol

Hindi Abstract
