# Supplementary material for: Functional precision approach in patients with very high risk acute lymphoblastic leukaemia in India: a single-centre cohort study
Source: Lancet Reg Health Southeast Asia. 2026 Jan 2;44:100710. doi: 10.1016/j.lansea.2025.100710 (PMC12805335; doi:10.1016/j.lansea.2025.100710)
Supplement: Supplemental Data [file mmc1.docx]

**Supplemental Methods**

Patients and treatment protocol: based initially on a combination of National Cancer Institute criteria, bulk of disease, CNS status, cytogenetics, and early response to prednisolone, which was assessed by peripheral blood blast counts on day 8 of induction. Patients with T-ALL were categorized as HR. SR and IR patients with MRD ≥0·01% (MRD positive) at end of induction (EoI) were escalated to high-risk post-induction. Patients with B-cell precursor (BCP) ALL were categorized into standard (SR), intermediate (IR), and high-risk (HR) groups as previously described ^1^.

Patient samples and biobanking: Blast percentage was recorded and mononuclear cellular fractions obtained by density centrifugation. These were either used fresh for DRP or cryopreserved for later use.

Cell culture: Immortalized h-TERT mesenchymal stromal cells (MSCs) were cultured in RPMI-Glutamax (Gibco, 61870-036) supplemented with 10% FBS and 1µM hydrocortisone (Gibco, H0888) in 37°C, 5% CO_2_.

Image-based Drug Response Profiling: h-TERT immortalised mesenchymal stromal cells (MSCs) were seeded at a density of 2·5 x 10^3^ cells/well in 384 well plates (Greiner, Cat No. 781090) in 30 mL AIM-V media (Gibco) 24hrs before adding 1 - 2·5 x 10^4^ primary ALL cells/well (depending on total cell number available) were added on the MSCs in 30μL of AIM-V and incubated for an additional 24h. Primary cells were seeded over hTERT-immortalised MSCs to mimic the bone marrow microenvironment ^2^. Cryo-preserved primary ALL cells were thawed in pre-warmed water bath at 37°C and 9ml of RPMI medium supplemented with 20% FBS was added slowly to the primary cell suspension. Cells were then spun down (Eppendorf, 5810R) in 300g for 5 minutes, supernatant discarded, and cell pellet re-suspended in AIM-V medium. The cell number and viability of the MSCs and ALL cells was determined before seeding with Trypan blue exclusion method. For samples with blasts less than 70%, flowcytometry based sorting (CD10^+^19^+^ for BCP ALL and CD5^+^CD7^+^ for T-ALL) was performed to enrich the leukemic population prior to seeding. Stock solutions for all drugs used in DRP were prepared at 5 mM concentration in DMSO, except for asparaginase, which was reconstituted in fresh frozen plasma (FFP) to a concentration of 500 IU/mL, and were stored at -80°C. On Day 3 of assay (after 24 hrs of co-culture incubation at 37°C, 5% CO_2_), six serial dilutions of compounds in the drug panel were prepared in AIM-V media and added in triplicates (10μL per well) The concentration range used for each drug was physiological ^3^. After 72h of incubation with compounds, CyQUANT (Life Technologies, C35011) live cell stain (nuclear stain 1:600, background suppressor 1:40) was added and incubated for 1h at 37°C, 5% CO_2_. Automated imaging was performed using the ImageXpress microscope (Molecular Devices) with 10x Plan Fluor objective with 0·3 NA (Nikon) (covering 50% of the well surface). Images were analyzed and live ALL cells counted using machine learning based workflow (Single-cell technologies; the DB, Finnish Institute of molecular medicine, FIMM)^4^. Idarubicin was taken as positive control and untreated wells as negative control. Post-assay live cell count of at least 1000 cells was considered as quality control passed and taken forward for response assessment. Drug compounds used for DRP were ordered from NIH Open Chemicals Repository ^5^.

Drug response quantification and analysis: Hierarchical clustering using Euclidean distance and complete‑linkage agglomeration was applied to DSS values to group patients and drug compounds with similar sensitivity patterns. Differential drug responses of patient groups of interest were evaluated by using the Mann-Whitney U test. Spearman’s rank correlation was used to assess the monotonic association between ex-vivo DSS and MRD levels. Receiver operating characteristic (ROC) analysis was performed in GraphPad Prism (version 10.0.2) and the pROC package in R to assess the discriminatory ability of MRD response (≥0·01%). In Prism, the area under the curve (AUC) is tested against the null hypothesis of AUC = 0.5 (no discriminative ability) by comparing the observed AUC with 0.5 and estimating the probability of obtaining such a result under the null. In R, AUCs and their 95% confidence intervals were estimated using the nonparametric DeLong method ^6^, and statistical significance versus AUC = 0.5 was assessed with the DeLong test. Optimal cut-off values were determined using Youden’s index ^7^, and 95% confidence intervals for sensitivity and specificity were calculated using the Wilson–Brown method ^8^.

References:

1. Gogoi MP, Das P, Das N, et al. Risk stratified treatment for childhood acute lymphoblastic leukaemia: a multicentre observational study from India. *Lancet Reg Health Southeast Asia*. Jun 2025;37:100593. doi:10.1016/j.lansea.2025.100593

2. Frismantas V, Dobay MP, Rinaldi A, et al. Ex vivo drug response profiling detects recurrent sensitivity patterns in drug-resistant acute lymphoblastic leukemia. *Blood*. Mar 16 2017;129(11):e26-e37. doi:10.1182/blood-2016-09-738070

3. Jamaladdin N, Sigaud R, Kocher D, et al. Key Pharmacokinetic Parameters of 74 Pediatric Anticancer Drugs Providing Assistance in Preclinical Studies. *Clin Pharmacol Ther*. Oct 2023;114(4):904-913. doi:10.1002/cpt.3002

4. Mund A, Coscia F, Kriston A, et al. Deep Visual Proteomics defines single-cell identity and heterogeneity. *Nat Biotechnol*. Aug 2022;40(8):1231-1240. doi:10.1038/s41587-022-01302-5

5. Developmental Therapeutics Program (DTP). National Cancer Institite. Accessed April 24, 2025, <https://dtp.cancer.gov/>

6. DeLong ER, DeLong DM, Clarke-Pearson DL. Comparing the areas under two or more correlated receiver operating characteristic curves: a nonparametric approach. *Biometrics*. Sep 1988;44(3):837-45.

7. Youden WJ. Index for rating diagnostic tests. *Cancer*. Jan 1950;3(1):32-5. doi:10.1002/1097-0142(1950)3:1<32::aid-cncr2820030106>3.0.co;2-3

8. Brown LD, Cai TT, DasGupta A. Interval Estimation for a Binomial Proportion. *Statistical Science*. 2001;16(2)doi:10.1214/ss/1009213286

| **Supplemental Table S1: Panel of clinical compounds used for DRP** | | | | |
| --- | --- | --- | --- | --- |
|  |  |  |  |  |
|  | **Drug name** | **Drug class** | **Drug range** |  |
| 1 | Prednisolone | Immunomodulator | 1-100000 nM |  |
| 2 | Vincristine | Mitotic inhibitor | 0.1-10000 nM |  |
| 3 | Daunorubicin | DNA damaging agent | 0.1-10000 nM |  |
| 4 | Asparaginase | Metabolic inhibitor | 0.01-1000 IU/L |  |
| 5 | Bortezomib | Proteosomal inhibitor | 0.001-100 nM |  |
| 6 | Venetoclax | Pro-apoptotic | 0.1-10000 nM |  |
| 7 | Panobinostat | Epigenetic modulator | 0.1-10000 nM |  |
| 8 | Mitoxantrone | DNA damaging agent | 0.001-100 nM |  |
| 9 | Cyclophosphamide | Alkylating agent | 0.1-10000 nM |  |
| 10 | Cytarabine | Purine analog | 0.1-10000 nM |  |
| 11 | Selinexor | Nuclear export inhibitor | 0.1-10000 nM |  |
| 12 | 6-thioguanine | Purine analog | 0.1-10000 nM |  |
| 13 | Idarubicin | DNA damaging agent | 0.1-10000 nM | Positive control |

| **Supplemental Table S2: Survival data of ALL risk groups treated on ICiCLe-ALL-14*** | | | | | |
| --- | --- | --- | --- | --- | --- |
| **Final Risk** | **N** | **TRD** | **Relapse + Disease progression** | **3-year EFS (95%CI)** | **3-year OS (95% CI)** |
| Standard | 212 | 5 | 56 | 71·3 (64-78) | 94 (89-97) |
| Intermediate | 153 | 4 | 36 | 67·1 (58-75) | 78·1 (70-84) |
| High | 182 | 18 | 40 | 76·7 (70-82) | 85·7 (80-90) |
| T | 95 | 1 | 13 | 80·9 (71-88) | 85 (76-91) |
| Very high | 48 | 2 | 32 | 38·1 (24-52) | 57·4 (41-71) |
|  |  |  |  |  |  |
| p-value |  |  |  | p < 0·0001 | p < 0·0001 |

TRD, treatment related deaths

*690 patients, excluding 25 very high-risk patients who received modified treatment

| **Supplemental Table S3: Patient Characteristics by MRD Group** | | | | | |
| --- | --- | --- | --- | --- | --- |
|  |  |  |  |  |  |
| **MRD Group** | **N** | **Age ≥10y** | **WCC ≥50 x10^9^/L** | **PPR** | **HRG** |
| Group 1 | 395 | 69 | 93 | 34 | 19 |
| Group 2 | 112 | 22 | 22 | 13 | 11 |
| Group 3 | 30 | 8 | 13 | 3 | 5 |
| **Pairwise p-values** | | | | | |
| **Comparison** |  | **Age ≥10y** | **WCC ≥50 x10^9^/L** | **PPR** | **HRG** |
| Group 2 vs Group 1 |  | 0.5797 | 0.3745 | 0.3565 | 0.0665 |
| Group 3 vs Group 1 |  | 0.2196 | **0.0243** | 0.7368 | **0.0203** |

WCC, presenting white cell count; PPR, prednisolone poor response; HRG, high risk cytogenetics

| **Supplemental Table S4: Survival outcomes based on MRD status at End of Induction (EoI) and End of Consolidation (EoC)** | | | | | | |
| --- | --- | --- | --- | --- | --- | --- |
| MRD Group | EoI MRD (%) | EoC MRD (%) | N | No of events | p value | Hazard ratio and 95% CI |
| 1 | <0·01 | <0·01 | 395 | 115 |  |  |
| 2 | ≥0·01 | <0·01 | 112 | 32 | p=0·364 | 1·2 (0·8-2) |
| 3 | ≥0·01 | ≥0·01 | 30 | 21 | p<0·001 | 3·65 (2-6) |

3 patients with persistent extramedullary disease were excluded.

| **Supplemental Table S5: Patient characteristics of samples used for drug response profiling** | | |
| --- | --- | --- |
|  |  | |
| Total samples | 112 | |
|  | N | % |
|  |  |  |
| **Immunophenotype** |  |  |
| B | 95 | 85 |
| T | 15 | 13 |
| Mixed | 2 | 2 |
|  |  |  |
| **Disease stage** |  |  |
| Newly diagnosed | 72 | 64 |
| First relapse | 34 | 30 |
| Second relapse | 6 | 5 |
|  |  |  |
| **Blasts (%)** |  |  |
| <50 | 1 | 1 |
| 50-70 | 16 | 14 |
| >70 | 95 | 85 |
| Mean (±SD) | 80 (±18) |  |
|  |  |  |
| **Viability at time of seeding (%)** |  |  |
| Mean (±SD) | 85 (±14·5) |  |

**Supplemental Table S7: Characteristics of patients with VHR ALL very high risk ALL treated with standard and modified delayed intensification (DI)**

**Supplemental Figure S1: Survival outcomes of all risk groups of ALL treated at Tata Medical Center,** **August 2013 and May 2023.**

Kaplan–Meier curves were plotted representing (A) 3-year event-free survival and (B) 3-year overall survival for each risk group of patients with ALL treated on ICiCLe-ALL-2014 protocol. The numbers of patients at risk are shown beneath the graph. Each risk group is represented by a different colour. *P* value was calculated using log-rank test.

**Supplemental Figure S2: Survival of VHR ALL is inferior survival to other risk groups.**

Kaplan–Meier curves were plotted representing (A) 3-year event-free survival and (B) 3-year overall survival for Non-VHR and VHR risk group of patients with ALL treated on ICiCLe-ALL-2014 protocol. The numbers of patients at risk are shown beneath the graph. Each risk group is represented by a different colour. *P* value was calculated using log-rank test.

**Supplemental Figure S3: Event-Free Survival (EFS) stratified by end-of-induction (EoI) and end-of-consolidation (EoC) MRD status**

Kaplan-Meier plot illustrating event-free survival in patients stratified by EoI and EoC minimal residual disease (MRD) status. Group 1 : MRD <0.01% at both EoI and EoC (n = 395); Group 2 : MRD ≥0.01% at EoI but <0.01% at EoC (n = 112); Group 3 : MRD ≥0.01% at both EoI and EoC (n = 30)

The number of patients at risk at each timepoint is shown below the x-axis. Each risk group is represented by a different colour. *P* value was calculated using log-rank test.

**Supplemental Figure S4. Ex vivo drug sensitivity in B-ALL versus T-ALL samples.**


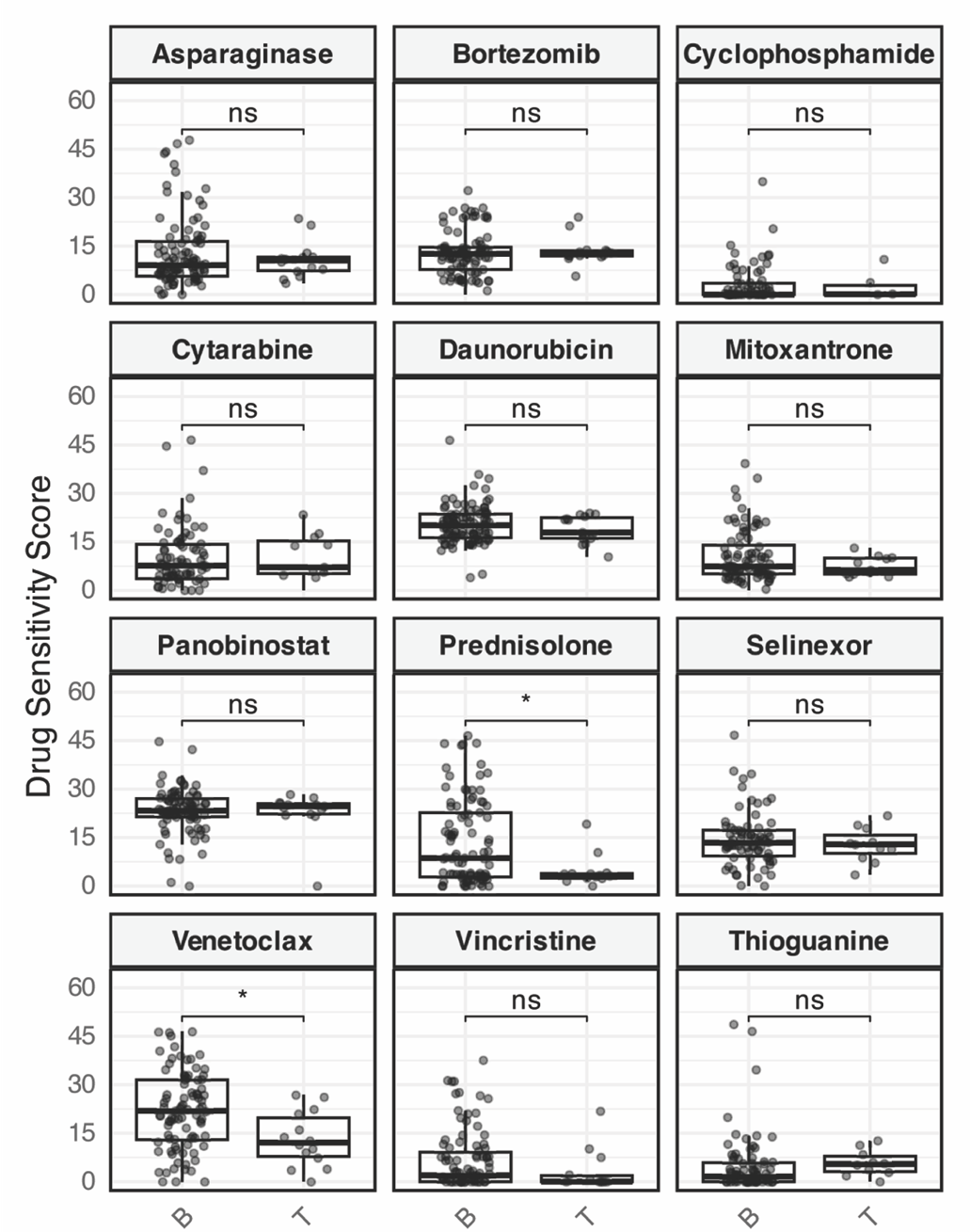


Boxplots comparing Drug Sensitivity Scores (DSS) for 13 agents between B-lineage (B) and T-lineage (T) ALL samples. Each dot represents an individual patient sample. Boxes represent the interquartile range (IQR), with horizontal lines indicating the median. Prednisolone and Venetoclax showed significantly higher DSS in B-ALL samples compared to T-ALL (* indicates p < 0.05), while no significant differences (ns) were observed for the remaining agents.

IPT, immunophenotype

**Supplemental Figure S5: Drug response profiling identifies patterns of sensitivity within cytogenetic subtypes of ALL.**

**
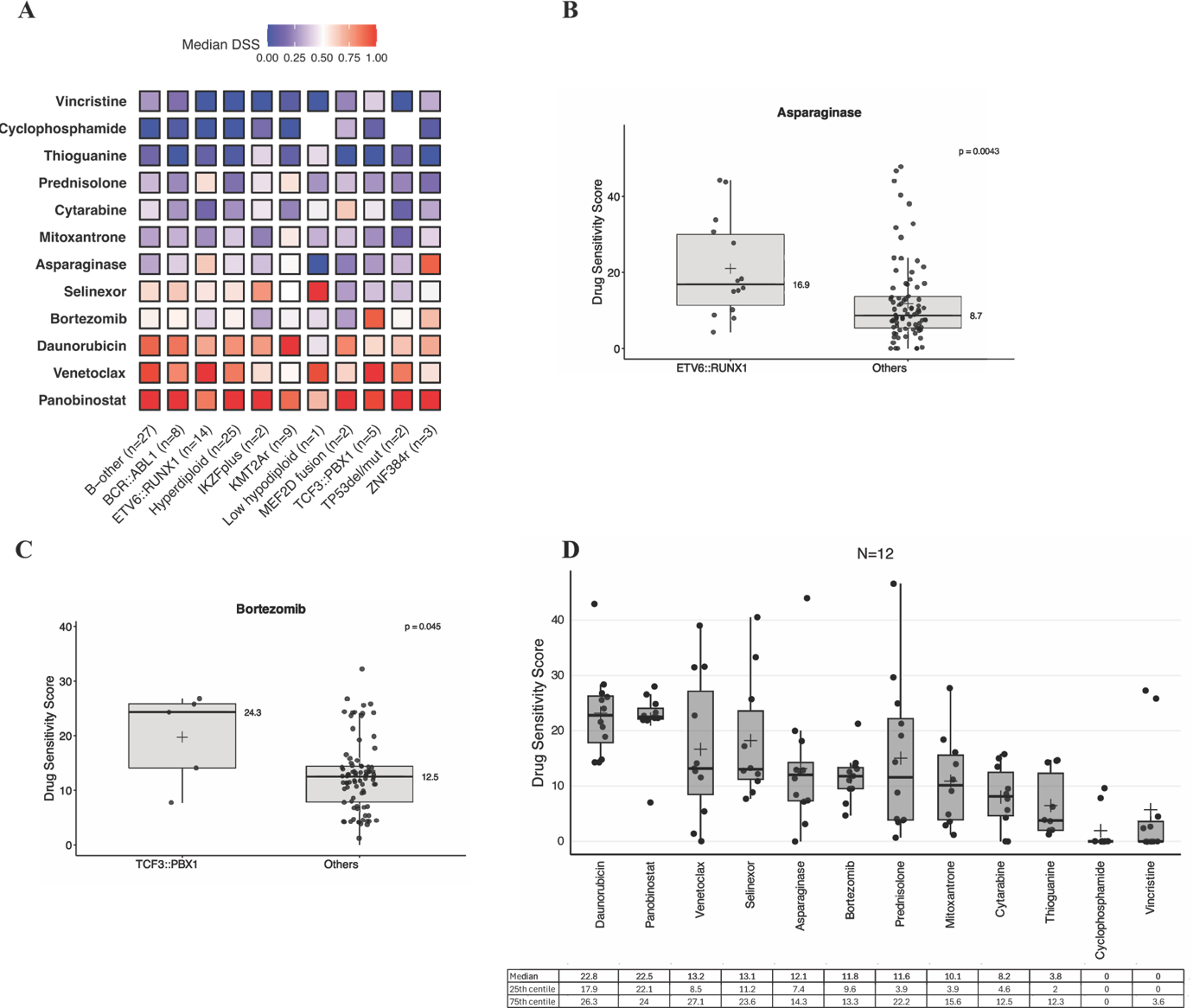
**

(A) Heatmap showing normalized median Drug Sensitivity Scores (DSS) across cytogenetic subtypes of ALL. Each square represents the median DSS for a specific drug within a given cytogenetic group, with values scaled from 0 to 1 within each cytogenetic group. The colour gradient indicates the magnitude of the scaled median DSS, where blue represents lower sensitivity, red represents higher sensitivity, and white is in between. Cytogenetic subtypes are labelled along the x-axis, followed by the sample size for each group in parentheses. The y-axis lists the drugs, and the legend at the top of the plot shows the scaled median DSS values. White represents missing or unavailable DSS data.

(B,C) Boxplots comparing Drug Sensitivity Scores (DSS) for Asparaginase and Bortezomib in *ETV6::RUNX1* (B) and *TCF3::PBX1* (C) samples respectively as compared to other cytogenetic subgroups. Each dot represents an individual patient sample. Boxes represent the interquartile range (IQR), with horizontal lines indicating the median and ‘+’ indicating mean DSS. Mann Whitney U test is for pairwise comparisons.

(D) Boxplots showing Drug Sensitivity Scores (DSS) for 12 samples with VHR genetics. Each dot represents an individual sample. Boxes indicate interquartile range (IQR), with whiskers representing 1.5× IQR. Horizontal lines within boxes represent median and “+” represents mean DSS values. Table shows median DSS, 25^th^ centile and 75^th^ centile for each drug.

**Supplemental Figure S6: Outcomes of patients with VHR ALL**

1. Flowchart representing events in patients with VHR ALL treated on standard delayed intensification (DI) or modified DI. TRM, treatment related mortality (B) Kaplan–Meier curve representing 1-year event-free survival of VHR ALL treated on modified DI with or without subsequent blinatumomab. The numbers of patients at risk are shown beneath the graph. *P* value was calculated using log-rank test.

**References for “Research in Context”**

1. Pemovska T, Kontro M, Yadav B, et al. Individualized systems medicine strategy to tailor treatments for patients with chemorefractory acute myeloid leukemia. *Cancer Discov* 2013; **3**(12): 1416-29.

2. Snijder B, Vladimer GI, Krall N, et al. Image-based ex-vivo drug screening for patients with aggressive haematological malignancies: interim results from a single-arm, open-label, pilot study. *Lancet Haematol* 2017; **4**(12): e595-e606.

3. Kornauth C, Pemovska T, Vladimer GI, et al. Functional Precision Medicine Provides Clinical Benefit in Advanced Aggressive Hematologic Cancers and Identifies Exceptional Responders. *Cancer Discov* 2022; **12**(2): 372-87.

4. Malani D, Kumar A, Brück O, et al. Implementing a Functional Precision Medicine Tumor Board for Acute Myeloid Leukemia. *Cancer Discovery* 2022; **12**(2): 388-401.

5. Lee SHR, Yang W, Gocho Y, et al. Pharmacotypes across the genomic landscape of pediatric acute lymphoblastic leukemia and impact on treatment response. *Nature Medicine* 2023; **29**(1): 170-9.

6. Frismantas V, Dobay MP, Rinaldi A, et al. Ex vivo drug response profiling detects recurrent sensitivity patterns in drug-resistant acute lymphoblastic leukemia. *Blood* 2017; **129**(11): e26-e37.

7. Place AE, Karol SE, Forlenza CJ, et al. Venetoclax Combined With Chemotherapy in Pediatric and Adolescent/Young Adult Patients With Relapsed/Refractory Acute Lymphoblastic Leukemia. *Pediatr Blood Cancer* 2025; **72**(6): e31630.

8. Short NJ, Jabbour E, Jain N, et al. A phase 1/2 study of mini-hyper-CVD plus venetoclax in patients with relapsed/refractory acute lymphoblastic leukemia. *Blood Adv* 2024; **8**(4): 909-15.

9. Zhang L, Zhang Z, Lu A, et al. Venetoclax-Based Regimen in Refractory or Relapsed Pediatric Acute Lymphoblastic Leukemia. *Acta Haematol* 2025: 1-7.

**Expanded Figure Legends**

**Figure 1. Patient risk stratification and treatment assignment for children with acute lymphoblastic leukaemia (ALL)**

Flow diagram illustrating risk stratification for 715 children diagnosed with ALL. Following 17 induction deaths, 698 patients, 591 B-cell precursor ALL (BCP ALL) and 106 T-ALL/MPAL, were stratified at end of induction into Standard Risk (SR), Intermediate Risk (IR), High Risk (HR), or Very High Risk (VHR) categories. A total of 73 patients were classified as VHR based on genetic features or poor therapeutic response, and either continued on the HR arm (n=48) or received modified treatment (n=25).

EoI, end of induction; EoC, end of consolidation; DI, delayed intensification

**Figure 2. Ex vivo drug response profiling reveals heterogeneous sensitivity patterns across clinical subgroups in ALL.**

1. Schematic representation of the drug response profiling (DRP) workflow. On Day 1, 2,500 MSCs were seeded per well in 384-well plates in AIM-V medium. On Day 2, 10,000–25,000 primary ALL cells were added per well onto MSCs. On Day 3, chemotherapeutic agents were added in serial dilutions in triplicate. On Day 6, wells were stained with the live-cell dye CyQUANT and imaged using automated microscopy. On Day 7, AI/ML-based image analysis quantified viable ALL cells and drug sensitivity scores (DSS) were calculated based on the area under the dose–response curve. Schematic created with BioRender.com.
2. Hierarchical clustering heatmap of DSS values across 112 patient samples and 12 drugs. Rows represent drugs; columns represent patient samples. Annotation bars show clinical risk category, immunophenotype and time point. Colour scale represents DSS from 0 (no sensitivity) to 40 (maximum sensitivity). Grey represents missing values. Four major response clusters (I–IV) were identified. The MSC-only heatmap is displayed on the right.
3. Comparison of DSS between group 1 (SR/IR) and group 2-4 (HR/VHR/relapse). Boxplots display DSS distribution for each drug. Mann Whitney U test is used for pairwise comparisons; *p* < 0·05 considered significant. * indicates *p* < 0·05; ** indicates *p* < 0·01, ns indicates not significant differences.
4. Comparison of DSS between newly diagnosed and relapsed ALL patients. Boxplots show drug-wise DSS variation across the two time-points. Mann Whitney U test is for pairwise comparisons; *p* < 0·05 considered significant. * indicates *p* < 0·05; ** indicates *p* < 0·01, ns indicates not significant differences.

MSCs, mesenchymal stromal cells; DSS, drug sensitivity score; MPAL, mixed-phenotype acute leukaemia

**Figure 3. Ex vivo drug sensitivity in samples from patients with Relapse/VHR ALL.**
Boxplots showing Drug Sensitivity Scores (DSS) across 67 samples from patients with relapsed/VHR ALL. Each dot represents an individual sample. Boxes denote interquartile range (IQR), with whiskers represent 1·5× IQR, horizontal lines within boxes indicate median values and “+” represents mean DSS. Standard chemotherapy agents - including prednisolone, cytarabine, thioguanine, vincristine, and cyclophosphamide - demonstrated limited ex vivo activity in this cohort (median DSS <10). In contrast, panobinostat and venetoclax showed the highest activity (median DSS 23·35 and 20·70, respectively), followed by daunorubicin (17·85), selinexor (12·65), and bortezomib (12·10). A summary table below the plot provides median DSS and corresponding IQR for each drug.

**Figure 4: Modification of delayed intensification (DI) block based on findings of drug response profiling improves response and survival rates in VHR ALL.**

1. Schematic of modified DI block. Agents highlighted in purple are the new drugs. Bortezomib (4 doses each 1·3mg/m^2^, on day 1, 4, 15 and 18) replaced vincristine. Venetoclax (360mg/m^2^/day) was added for 3 weeks (day 1-21). Bone marrow response assessment was done at end of 6 weeks (subject to count recovery) from the start of modified DI. The schematic shows the full schedule, including doses and timing of dexamethasone, PEG-asparaginase, mitoxantrone, cyclophosphamide, cytarabine, 6-mercaptopurine, and intrathecal (IT) methotrexate.
2. Decision-making algorithm for eligibility for modified DI. VHR criteria, general condition, and DRP results were reviewed by a leukaemia tumour board. Patients meeting criteria and deemed fit for intensified therapy were offered modified DI following informed consent. Patients ineligible for modification received conventional DI.
3. Longitudinal MRD response in VHR patients treated with modified DI. Each line represents an individual patient’s MRD trajectory at EoI, EoC, EoIM and EoDI. Green lines indicate MRD reduction, including 8 patients achieving MRD negativity (<0·01%). Red lines indicate poor response (MRD ≥0·01% post modified-DI) or progression. For poor responders, outcomes including survival, HSCT, and blinatumomab exposure are annotated on the graph.
4. Landmark analysis comparing event-free survival (EFS) between modified DI and standard DI in VHR ALL. Kaplan–Meier curves show superior EFS for the modified DI group (81·8% [58–93]) compared with standard DI (67·7% [49–81]). The log-rank test yielded *p* = 0·0324. Numbers at risk at each timepoint are displayed below the plot.

DRP, drug response profiling; HSCT, hematopoietic stem cell transplant; EoI, end of induction; EoC, end of consolidation; EoIM, end of interim maintenance; EoModifiedDI, end of modified DI; blina, blinatumomab.

**Figure 5: Correlating ex-vivo drug sensitivity (venetoclax, bortezomib) with clinical response to modified delayed intensification.**

(a, b) Correlation between Drug Sensitivity Scores (DSS) and end-of-modified DI (Eo-Modified DI) MRD levels. Scatter plots show DSS for Venetoclax (a) and Bortezomib (b) plotted against MRD on a logarithmic scale. Horizontal dashed lines indicate the MRD threshold of 0·01%; vertical dashed lines mark DSS thresholds for resistance (venetoclax: 8·85, bortezomib: 12·05). Each point represents an individual patient sample. Spearman’s correlation coefficient (ρ) and corresponding *p*-values are displayed, venetoclax exhibited a strong inverse correlation (ρ = –0·82; *p* = 0·007), whereas bortezomib showed no significant relationship (ρ = –0·07; *p* = 0·859).

(c, d) Receiver operating characteristic (ROC) curves evaluating DSS as a predictor of MRD response. Venetoclax (c) achieved an AUC of 1·00 (p < 0·0001), whereas bortezomib (d) yielded an AUC of 0·65 (p = 0·50). The ROC curves reflect sensitivity-specificity performance across DSS thresholds.

(e, f) Contingency matrices comparing DSS-based classification with clinical MRD response. For venetoclax (e), DSS ≥8·85 predicted MRD negativity with 100% sensitivity and 100% specificity (5/5 true negatives; 4/4 true positives). For bortezomib (f), DSS ≥12·05 correctly identified all MRD-positive patients (100% sensitivity) but showed limited specificity (2 false positives; specificity 60%).

STROBE Statement—checklist of items that should be included in reports of observational studies

|  | Item No. | Recommendation | Page  No. |
| --- | --- | --- | --- |
| **Title and abstract** | 1 | (*a*) Indicate the study’s design with a commonly used term in the title or the abstract | 1 |
|  |  | (*b*) Provide in the abstract an informative and balanced summary of what was done and what was found | 3 |
| Introduction | | | |
| Background/rationale | 2 | Explain the scientific background and rationale for the investigation being reported | 6 |
| Objectives | 3 | State specific objectives, including any prespecified hypotheses | 6 |
| Methods | | | |
| Study design | 4 | Present key elements of study design early in the paper | 7 |
| Setting | 5 | Describe the setting, locations, and relevant dates, including periods of recruitment, exposure, follow-up, and data collection | 7 |
| Participants | 6 | (*a*) *Cohort study*—Give the eligibility criteria, and the sources and methods of selection of participants. Describe methods of follow-up | 7,8 |
|  |  |  |  |
| Variables | 7 | Clearly define all outcomes, exposures, predictors, potential confounders, and effect modifiers. Give diagnostic criteria, if applicable | 8 |
| Data sources/ measurement | 8 | For each variable of interest, give sources of data and details of methods of assessment (measurement). Describe comparability of assessment methods if there is more than one group | 8 |
| Bias | 9 | Describe any efforts to address potential sources of bias | Not Applicable |
| Study size | 10 | Explain how the study size was arrived at | Not Applicable |

Continued on next page

| Quantitative variables | 11 | Explain how quantitative variables were handled in the analyses. If applicable, describe which groupings were chosen and why | 8 |  |
| --- | --- | --- | --- | --- |
| Statistical methods | 12 | (*a*) Describe all statistical methods, including those used to control for confounding | 8 |  |
|  |  | (*b*) Describe any methods used to examine subgroups and interactions | Not Applicable |  |
|  |  | (*c*) Explain how missing data were addressed |  |  |
|  |  | (*d*) *Cohort study*—If applicable, explain how loss to follow-up was addressed | Not Applicable |  |
|  |  | (*e*) Describe any sensitivity analyses | Not Applicable |  |
| Results | | | | |
| Participants | 13 | (a) Report numbers of individuals at each stage of study—eg numbers potentially eligible, examined for eligibility, confirmed eligible, included in the study, completing follow-up, and analysed | 10 |  |
|  |  | (b) Give reasons for non-participation at each stage | Not Applicable |  |
|  |  | (c) Consider use of a flow diagram | Figure 1 |  |
| Descriptive data | 14 | (a) Give characteristics of study participants (eg demographic, clinical, social) and information on exposures and potential confounders | Supplemental Table S2 |  |
|  |  | (b) Indicate number of participants with missing data for each variable of interest | Not Applicable |  |
|  |  | (c) *Cohort study*—Summarise follow-up time (eg, average and total amount) | 10 |  |
| Outcome data | 15 | *Cohort study*—Report numbers of outcome events or summary measures over time | 10 |  |
|  |  |  |  |  |
|  |  |  |  |  |
| Main results | 16 | (*a*) Give unadjusted estimates and, if applicable, confounder-adjusted estimates and their precision (eg, 95% confidence interval). Make clear which confounders were adjusted for and why they were included | 13-16 |  |
|  |  | (*b*) Report category boundaries when continuous variables were categorized | Not Applicable |  |
|  |  | (*c*) If relevant, consider translating estimates of relative risk into absolute risk for a meaningful time period | Not Applicable |  |

Continued on next page

| Other analyses | 17 | Report other analyses done—eg analyses of subgroups and interactions, and sensitivity analyses | 12-14 (DRP) |  |
| --- | --- | --- | --- | --- |
| Discussion | | | | |
| Key results | 18 | Summarise key results with reference to study objectives | 18-19 |  |
| Limitations | 19 | Discuss limitations of the study, taking into account sources of potential bias or imprecision. Discuss both direction and magnitude of any potential bias | 19 |  |
| Interpretation | 20 | Give a cautious overall interpretation of results considering objectives, limitations, multiplicity of analyses, results from similar studies, and other relevant evidence | 18-20 |  |
| Generalisability | 21 | Discuss the generalisability (external validity) of the study results | 20-21 |  |
| Other information | |  | | |
| Funding | 22 | Give the source of funding and the role of the funders for the present study and, if applicable, for the original study on which the present article is based | 22 |  |

**Note:** An Explanation and Elaboration article discusses each checklist item and gives methodological background and published examples of transparent reporting. The STROBE checklist is best used in conjunction with this article (freely available on the Web sites of PLoS Medicine at http://www.plosmedicine.org/, Annals of Internal Medicine at http://www.annals.org/, and Epidemiology at http://www.epidem.com/). Information on the STROBE Initiative is available at www.strobe-statement.org.
