# Supplementary material for: Functional precision approach in patients with very high risk acute lymphoblastic leukaemia in India: a single-centre cohort study
Source: Lancet Reg Health Southeast Asia. 2026 Jan 2;44:100710. doi: 10.1016/j.lansea.2025.100710 (PMC12805335; doi:10.1016/j.lansea.2025.100710)
Supplement: Treatment Protocol [file mmc2.docx]

**Protocol: Modified DI for Very High Risk Acute Lymphoblastic Leukaemia**

**Recommended schedule**

Blinatumomab

Cycle 1

Blinatumomab

Cycle 2

Modified DI

**BME + MRD**

Pre-Blina C1

**BME + MRD**

Post-Blina C1

Allo-SCT

**BME + MRD**

Post-Blina C2

B Cell

T Cell

|  | | | | |
| --- | --- | --- | --- | --- |
| **Phase** | **Drug** | **Route** | **Dose** | **Schedule** |
| **Modified DI** | Dexamethasone | Oral | 10 mg/m^2^ (max, 20 mg/day) | Days 1 – 7 ; 15 – 21 |
|  | Bortezomib | Intravenous | 1·3 mg/m^2^ | Days 1, 4 ; 15, 18 |
|  | Methotrexate | Intrathecal | Based on age | Days 1, 15 |
|  | Venetoclax | Oral | 360 mg/m^2^ (max, 400 mg/day) | Days 1 – 21 |
|  | PEG-asparaginase | Intramuscular | 1000 U/m^2^ | Days 1 |
|  | Mitoxantrone | Intravenous | 10 mg/m^2^ | Day 1 |
|  | Cyclophosphamide | Intravenous | 300 mg/m^2^ (12 hourly) | Days 29, 30 |
|  | Cytarabine | Intravenous | 75 mg/m^2^ | Days 30 – 33 ; 37 – 40 |
|  | 6-Mercaptopurine | Oral | 40 mg/m^2^ | Days 29 – 42 |
|  | Liposomal amphotericin B | Intravenous | 2·5 mg/kg | Twice a week, Week 1, 2, 3, 4 |
|  | Cotrimoxazole | Oral | Based on body surface area | 2 consecutive days, every week |
| **Blinatumomab** | Cycle 1 | Intravenous | 5 mcg/m^2^, continuous IV | Days 1 – 4 |
|  |  |  | 15 mcg/m^2^, continuous IV | Days 5 – 28 |
|  | Cycle 2 | Intravenous | 15 mcg/m^2^, continuous IV | Days 1 – 28 |
| **Allogeneic-stem cell transplantation** | | | | |

IV, intravenous; max, maximum; PEG-asparaginase, polyethylene-conjugated *E. coli* L asparaginase

Methotrexate dose for intrathecal treatment: Age < 2 years, 8 mg; Age ≥ 2 - < 3 years, 10 mg; ≥ 3 years, 12 mg

Blinatumomab, only for B cell-precursor VHR ALL; MRD evaluation & intrathecal treatment before each cycle; 14-day interval between cycles
