# Supplementary material for: Functional precision approach in patients with very high risk acute lymphoblastic leukaemia in India: a single-centre cohort study
Source: Lancet Reg Health Southeast Asia. 2026 Jan 2;44:100710. doi: 10.1016/j.lansea.2025.100710 (PMC12805335; doi:10.1016/j.lansea.2025.100710)
Supplement: Hindi Abstract [file mmc3.docx]

# सारांश

## पृष्ठभूमि

मेज़रेबल रेज़िडुअल डिज़ीज़ (MRD) का बने रहना और उच्च-जोखिम वाले साइटोजेनेटिक परिवर्तन, बचपन के तीव्र लसीकाणु ल्यूकेमिया (Acute Lymphoblastic Leukaemia, ALL) में बीमारी के दोबारा लौटने (रिलैप्स) के प्रमुख संकेतक माने जाते हैं।

## विधियाँ

अगस्त 2013 से मई 2023 के बीच एक केंद्र पर ICiCLe-ALL-2014 प्रोटोकॉल से उपचारित बच्चों के परिणामों का विश्लेषण किया गया। नैदानिक या पुनरावृत्त (रिलैप्स) नमूनों पर ex-vivo ड्रग रिस्पॉन्स प्रोफाइलिंग (DRP) की गई। अत्यधिक उच्च-जोखिम (Very High Risk, VHR) वाले मरीजों को DRP से प्राप्त परिणामों के आधार पर उपचार में संशोधन दिया गया। विभिन्न जोखिम समूहों में इवेंट-फ्री सर्वाइवल (EFS) और कुल सर्वाइवल (OS) की तुलना की गई।

## निष्कर्ष

कुल 715 मरीजों में, औसतन 55 महीनों के फॉलो-अप पर, 3-वर्षीय EFS क्रमशः मानक-जोखिम में 71%, मध्यम-जोखिम में 67%, उच्च-जोखिम में 77%, T-ALL में 81% और VHR समूह में 38% रही (p < 0·0001)। एंड-कंसॉलिडेशन पर MRD की निरंतर उपस्थिति कम EFS (40·3%, p < 0·0001) से जुड़ी थी।
112 नमूनों पर किए गए DRP से यह पाया गया कि पैनोबिनोस्टैट, वेनेटोक्लैक्स, डॉउनोरूबिसिन, सेलिनेक्सोर, और बॉर्टेज़ोमिब VHR या रिलैप्स ALL में प्रभावी दवाएँ हैं।
नवंबर 2020 से 25 VHR मरीजों को वेनेटोक्लैक्स और बॉर्टेज़ोमिब सहित संशोधित उपचार दिया गया। इस समूह में 1.5-वर्षीय EFS 81·8% रही, जबकि मानक उपचार समूह में यह 67·7% थी (p = 0·0324)। वेनेटोक्लैक्स की संवेदनशीलता MRD क्लियरेंस से महत्वपूर्ण रूप से संबंधित थी (p = 0·0070)।

## व्याख्या

DRP के उपयोग से VHR बाल ALL के इलाज में प्रभावी दवाओं की पहचान संभव हुई। वेनेटोक्लैक्स और बॉर्टेज़ोमिब को शामिल करने से उपचार सहनीय रहा और प्रारंभिक जीवितता में सुधार देखा गया। ये परिणाम दर्शाते हैं कि VHR ALL में DRP-आधारित उपचार रणनीतियों का आगे मूल्यांकन किया जाना चाहिए।

## वित्तपोषण

DBT-Wellcome India Alliance और Tata Consultancy Services द्वारा वित्तपोषित।

*Editorial disclaimer:*

*This translation in Hindi was submitted by the authors and we reproduce it as supplied. It has not been peer reviewed. Our editorial processes have only been applied to the original abstract in English, which should serve as reference for this manuscript.*”
